# A fully reversible 25-hydroxy steroid kinase involved in oxygen-independent cholesterol side-chain oxidation

**DOI:** 10.1016/j.jbc.2021.101105

**Published:** 2021-08-21

**Authors:** Christian Jacoby, Malina Goerke, Dominik Bezold, Henning Jessen, Matthias Boll

**Affiliations:** 1 Faculty of Biology, Albert-Ludwigs-Universität Freiburg, Freiburg, Germany; 2 Institute of Organic Chemistry, Albert-Ludwigs-Universität Freiburg, Freiburg, Germany

**Keywords:** cholesterol, kinase, steroid degradation, high-energy phosphoester, ADD, androsta-1,4-diene-3,17-dione, CDO, cholest-1,4-diene-3-one, CEO, cholest-4-en-3-one, DDO, desmost-1,4-diene-3-one, S25 DH_1_, steroid C25 dehydrogenase 1, UPLC, ultraperformance liquid chromatography

## Abstract

The degradation of cholesterol and related steroids by microbes follows fundamentally different strategies in aerobic and anaerobic environments. In anaerobic bacteria, the primary C26 of the isoprenoid side chain is hydroxylated without oxygen *via* a three-step cascade: (i) water-dependent hydroxylation at the tertiary C25, (ii) ATP-dependent dehydration to form a subterminal alkene, and (iii) water-dependent hydroxylation at the primary C26 to form an allylic alcohol. However, the enzymes involved in the ATP-dependent dehydration have remained unknown. Here, we isolated an ATP-dependent 25-hydroxy-steroid kinase (25-HSK) from the anaerobic bacterium *Sterolibacterium denitrificans*. This highly active enzyme preferentially phosphorylated the tertiary C25 of steroid alcohols, including metabolites of cholesterol and sitosterol degradation or 25-OH-vitamin D_3_. Kinetic data were in agreement with a sequential mechanism *via* a ternary complex. Remarkably, 25-HSK readily catalyzed the formation of γ-(^18^O)_2_-ATP from ADP and the C25-(^18^O)_2_-phosphoester. The observed full reversibility of 25-HSK with an equilibrium constant below one can be rationalized by an unusual high phosphoryl transfer potential of tertiary steroid C25-phosphoesters, which is ≈20 kJ mol^−1^ higher than that of standard sugar phosphoesters and even slightly greater than the β,γ-phosphoanhydride of ATP. In summary, 25-HSK plays an essential role in anaerobic bacterial degradation of zoo- and phytosterols and shows only little similarity to known phosphotransferases.

Cholesterol and related zoo-, phyto-, and mycosterols share a common architecture comprising a tetracyclic sterane core structure linked to an isoprenoid side chain. They are involved in maintaining membrane fluidity in eukaryotes and serve as precursor molecule for a large number of steroids with various endocrine and other physiological activities (1, 2). Microbial transformation/degradation of steroids alters the sex steroid profile of eukaryotic hosts and is essential for bacterial pathogens such as *Mycobacterium tuberculosis* to persist in macrophages (3, 4, 5, 6). Once released into the environment, their hydrophobicity makes steroids ubiquitous persistent pollutants of global environmental concern. Microbial biodegradation is a major process to eliminate endocrine-disrupting steroids from the environment (7, 8, 9).

In aerobic cholesterol degrading microorganisms, side chain degradation is initiated by oxidation of ring A (10, 11) and/or by cytochrome P450 monooxygenase-dependent hydroxylation and oxidation of the unactivated primary C26 to a C26 carboxylate (Fig. 1*A*), (12, 13). After activation of the latter to a CoA ester, the side chain is degraded by modified β-oxidation to acetyl-CoA, propionyl-CoA, and androsta-1,4-diene-3,17-dione (ADD), (14). In anaerobic bacteria, the cholesterol side chain is also degraded by β-oxidation *via* a C26 carboxylate intermediate (15). However, oxidation of primary C26 is achieved by an oxygenase-independent strategy that has been studied mainly in the denitrifying β-proteobacterial model organism *Sterolibacterium denitrificans* Chol-1S.

**Figure 1:**
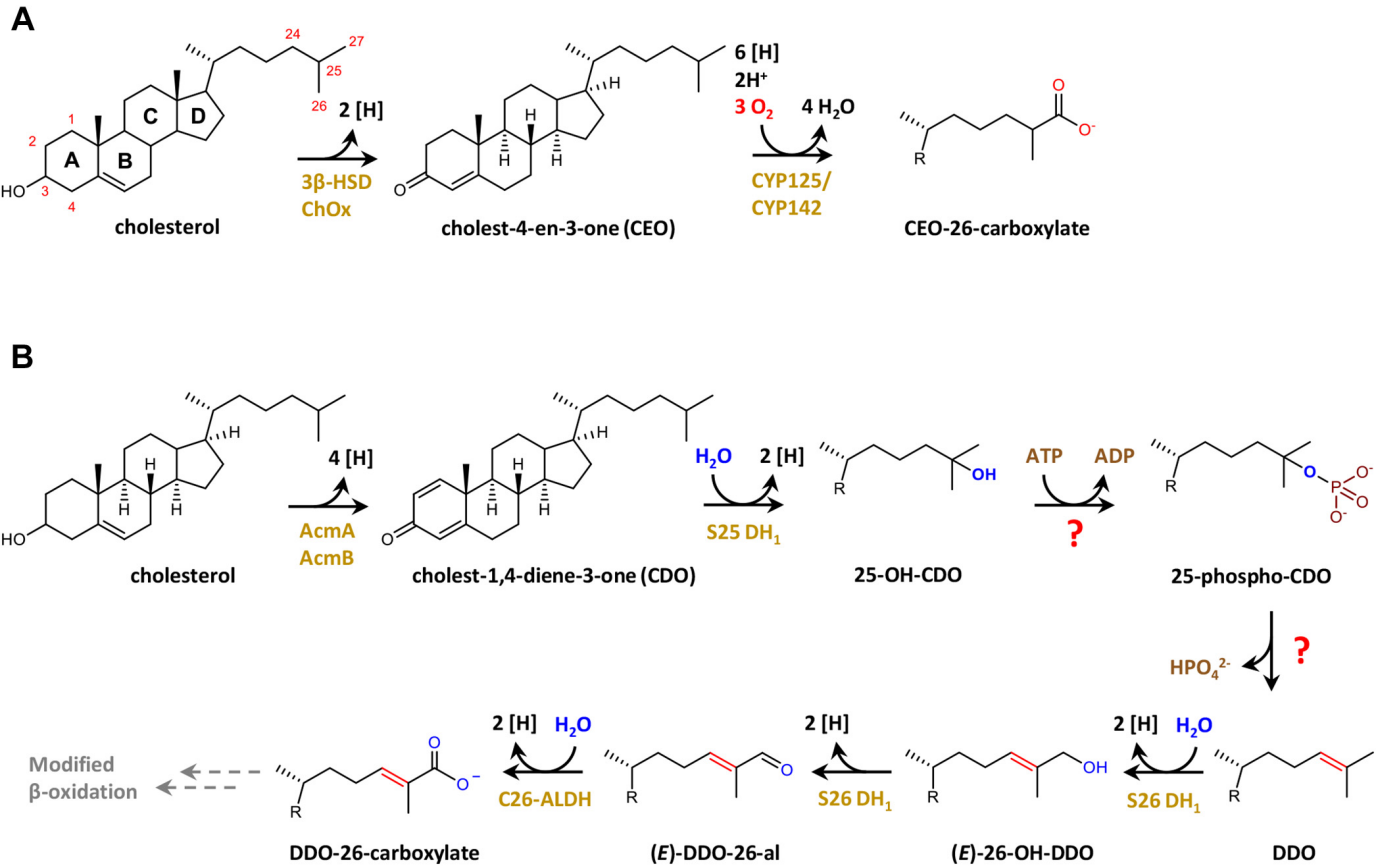
**Initial steps of microbial cholesterol side chain degradation.***A*, enzymes and intermediates involved in the transformation of cholesterol to cholest-4-en-3-one-26-carboxylate (CEO-26-carboxylate) in aerobic cholesterol degrading bacteria. *B*, oxygen-independent isoprenoid side chain activation to desmost-1,4-diene-3-one-26-carboxylate (DDO-26-carboxylate) as discovered in *S. denitrificans* grown with cholesterol and nitrate. C26-ALDH, (E)-DDO-26-al dehydrogenase

In *S. denitrificans*, cholesterol degradation is initiated by dehydrogenation of ring A to cholest-4-en-3-one (CEO) by a 3β-hydroxysteroid dehydrogenase (AcmA), which may be further dehydrogenated to cholest-1,4-diene-3-one (CDO) by 3-ketosteroid dehydrogenase (AcmB) (16, 17). Both CEO and CDO then serve as substrate for steroid C25 dehydrogenase 1 (S25 DH_1_) that uses water to hydroxylate the tertiary C25 atom to corresponding 25-OH-steroids (Fig. 1*B*). The enzyme is a member of the type II DMSO reductase family of molybdenum cofactor containing enzymes, and catalysis probably proceeds *via* hydride transfer from C25 with a carbocation intermediate (18, 19, 20). The genome of *S. denitrificans* contains eight homologous genes coding for S25 DH-like enzymes. S25 DH_1-4_ were identified to hydroxylate zoo- and phytosterols to tertiary alcohols at C25 (19).

Recently, we demonstrated in extracts of *S. denitrificans* grown with cholesterol and nitrate that the formation of a tertiary alcohol at the steroid side chain initiates an enzymatic reaction cascade that finally results in the formation of a C26-carboxylate. It comprises the ATP-dependent dehydration of 25-OH-CDO to the Δ24 alkene desmost-1,4-diene-3-one (DDO), followed by a second water-dependent hydroxylation at the allylic C26 giving (*E*)-26-OH-DDO (Fig. 1) (15). The latter step is catalyzed by steroid C26 dehydrogenase 1 (S26 DH_1_), a further molybdenum-dependent enzyme of the DMSO reductase family that also catalyzed the subsequent dehydrogenation of the allylic alcohol to the corresponding aldehyde. The C–H bond dissociation energy to carbocations and a hydride is significantly lower at the allylic C26 of DDO in comparison to the primary C26 of CDO or cholesterol. In summary, this unprecedented cascade allows for the hydroxylation of an unactivated primary carbon with water at the expense of one ATP (Fig. 1*B*) (15).

In agreement, *S. denitrificans* extracts converted the chemically synthesized 25-phospho-CDO to desmost-1,4-diene-3-one (DDO) demonstrating that it represents an intermediate during the ATP-dependent dehydration of 25-OH-CDO to DDO (15). It has remained unclear, whether the assumed phosphorylation and phosphate elimination are catalyzed by a single enzyme or by a distinct kinase and lyase. The gene(s) and enzyme(s) involved in the ATP-dependent dehydration of 25-OH-steroids have remained unknown.

In this work, we aimed at identifying the enzyme involved in formation of the assumed 25-phospho-CDO intermediate during anaerobic cholesterol degradation in the denitrifying β-proteobacterium *S. denitrificans*. For this purpose, a 25-phospho-CDO forming activity was enriched from *S. denitrificans* to allow for the identification of the encoding gene. After its heterologous production, we identified a highly active and specific 25-hydroxy-steroid kinase (25-HSK) that ATP-dependently formed the 25-phosphoester of CDO and related 25-OH-steroids with an extremely high phosphoryl group transfer potential.

## Results

### ATP-dependent dehydration of 25-OH-CDO involves a 25-hydroxy-steroid kinase

**Figure 2:**
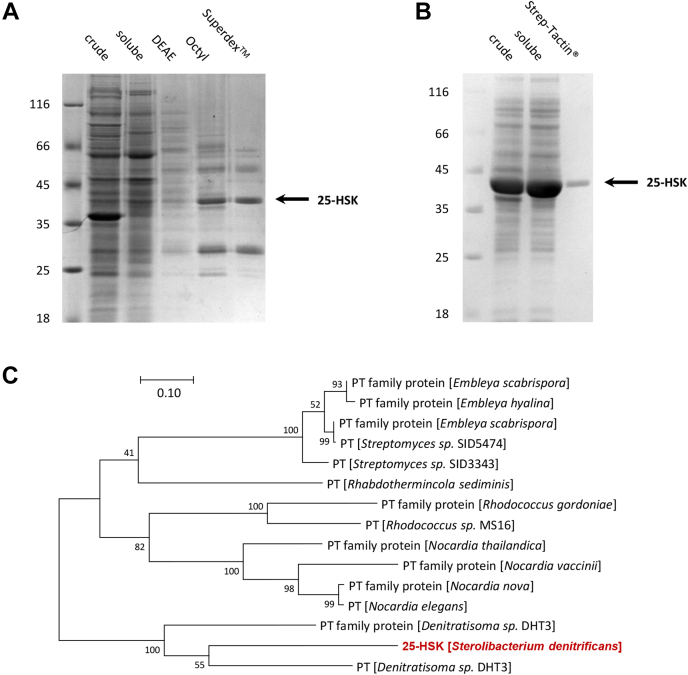
**SDS-PAGEanalysis of active pools during the enrichment of25-HSKfrom*****S. denitrificans*****wild type (*****A*****), during the enrichment of recombinant25-HSKfrom*****E. coli*****BL21 (*****B*****), and phylogenetic analysis of25-HSK-like proteins (*****C*****)**. *A*, crude: 20 μg crude extracts, soluble: 20 μg soluble proteins after centrifugation at 150,000*g*, DEAE: 10 μg protein after DEAE Sepharose chromatography, Octyl: 5 μg protein after Octyl-Sepharose chromatography, Superdex: 5 μg protein after size-exclusion chromatography at Superdex 10/300 GL. *B*, crude: 20 μg *E coli* crude extracts expressing SDENCHol_21286, soluble: 20 μg soluble proteins, Strep-Tactin: 2 μg after Strep-Tactin chromatography. The molecular size markers (kDa) are indicated. The *arrow* points to the protein band that was finally identified as 25-HSK. *C*, phylogenetic tree of selected 25-HSK related enzymes. The phylogenetic tree was constructed using the Maximum Likelihood method based on the Poisson correction model (1000 Bootstrap values). PT, phosphotransferase.

To identify the unknown enzyme(s) involved in the ATP-dependent conversion of 25-OH-CDO to DDO, we opted for an enrichment from soluble extracts of *S. denitrificans* cells grown with cholesterol and nitrate as carbon and energy sources. Activity was monitored in an ultraperformance liquid chromatography (UPLC) based assay following the ATP-dependent consumption of 25-OH-CDO and formation of product(s) (Fig. S1). After a first DEAE anion exchange chromatography enrichment step, protein fractions were obtained that converted 25-OH-CDO (0.5 mM) to 25-phospho-CDO as the only product in the presence of 2-hydroxypropyl-β-cyclodextrin (HPCD, solubility enhancer) and MgATP (5 mM) (Fig. S1). This finding indicates that the ATP-dependent dehydration of 25-OH-CDO involves two distinct activities, a kinase activity henceforth referred to as 25-hydroxy-steroid kinase (25-HSK) and a phosphate eliminating C–O lyase. Further enrichment of the kinase activity *via* hydrophobic interaction chromatography and size-exclusion chromatography yielded two major protein bands migrating at 30 and 40 kDa in SDS–polyacrylamide gels and a minor one migrating at 50 kDa (Fig. 2*A*). The final 25-HSK activity enrichment factor was 117 with a yield of 8% (Table 1).

**Table 1:** Enrichment of 25-HSK from 10 g (wet weight) *S. denitrificans* cells

Fraction	Protein (mg)	Specific activity (nmol min^−1^ mg^−1^)	Enrichment factor	Yield (%)
Crude extract	1050	8 ± 0.5		
Soluble proteins	291	11 ± 2	1.4	100
DEAE-Sepharose	47	30 ± 4	3.8	44
Octyl-Sepharose	3.5	200 ± 25	25	21
Superdex 200	0.3	940 ± 25	117	8

The numbers refer to the mean values ± standard deviation of three independent replicates.

Mass spectrometric analyses of tryptic peptides from the three dominant enriched protein bands identified proteins from *S. denitrificans* with highest scores to acyl-CoA synthetases (60 kDa band), phytoene dehydrogenases (50 kDa), phosphotransferases (40 kDa band), and luciferases (30 kDa band, Table S1). Thus, the 40 kDa protein is the most likely candidate responsible for 25-OH steroid phosphorylation. In a previous differential proteome analysis, the corresponding gene product (SDENChol_21286, *S. denitrificans* genome annotation, A0A7Z7HU23) was upregulated in cells grown with cholesterol *versus* cells grown with testosterone that lacks the isoprenoid side chain (21). Protein blast analysis of the protein with the NCBI database (refseq_protein) showed highest similarities to gene products from the 17β-estradiol-degrading *Denitratisoma* sp. DHT3 (55% and 51% amino acid sequence identity, respectively, accession numbers WP_186453974.1 and WP_145841310.1) (Fig. 2*C*, Table S2). High similarities (≤46% identity) were also found to putative phosphotransferase family gene products from Actinobacteria.

### Heterologous expression of the encoding gene yields a highly active 25-HSK

The putative gene encoding 25-HSK (WP_154717265.1) was cloned and expressed in *E. coli* BL21 as C-terminal Strep-tagged fusion protein. Extracts from *E. coli* BL21 expressing this gene catalyzed 25-OH-CDO phosphorylation with a specific activity of 0.9 ± 0.05 μmol min^−1^ mg^−1^, whereas cells containing the same plasmid without the gene showed no such activity (<0.001 μmol min^−1^ mg^−1^). After enrichment by Strep-Tactin affinity chromatography, SDS-PAGE analysis of the active protein pool revealed a single protein band with an apparent molecular mass of approximately 40 kDa (Fig. 2*B*) fitting to the mass (43.3 kDa) deduced from the amino acid sequence. The native molecular mass of recombinant 25-HSK was determined by gel filtration and was 45 ± 5 kDa, indicating a monomeric architecture. The 25-HSK preparation catalyzed the time-dependent transformation of 1 mM 25-OH-CDO to 25-phospho-CDO in the presence of 5 mM ATP and 10 mM MgCl_2_ at a specific activity of 19.5 ± 0.5 μmol min^−1^ mg^−1^ (Fig. S2). The enzyme was used for all kinetic experiments and was stored in 50 mM MOPS/KOH pH 7 buffer for several months at 4 °C without a significant loss of activity.

### 25-HSK prefers tertiary C25 alcohols of steroids

**Figure 3:**
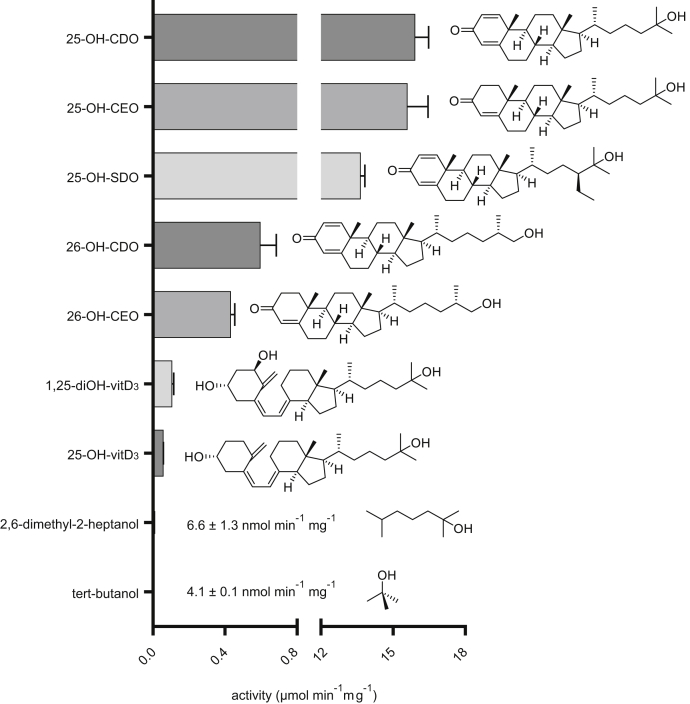
**Relative specific activities of 25-HSK with various tertiary and primary alcohols.** Activities were performed by a coupled photometric assay in presence of 0.5 mM substrate and 5 mM ATP. The error bars refer to the standard deviation of three independent replicates.

For testing the substrate preference of 25-HSK, a photometric enzyme assay was used that coupled ATP hydrolysis to the reduction of NADH to NAD^+^ in the presence of phosphoenolpyruvate, pyruvate kinase, and lactate dehydrogenase. The highest specific activity was observed with 25-OH-CDO (0.5 mM) at 16 ± 0.6 μmol min^−1^ mg^−1^ fitting well to the activity determined in UPLC-based assays (Fig. 3). Only slightly lower values were determined with other C25 alcohols of steroids with modifications in ring A or in the isoprenoid side chain. 25-OH-steroids with other modifications were converted at lower rates such as 25-hydroxy-vitamin D_3_ (VitD_3_). Nonsteroidal tertiary alcohols were converted albeit only at <0.1% of the activity of 25-OH-CDO. Notably, low but significant activities were also detected for primary 26-OH-steroids such as (25R)-26-OH-CDO and (25R)-26-OH-CEO.

### 25-HSK reaction is fully reversible

**Figure 4:**
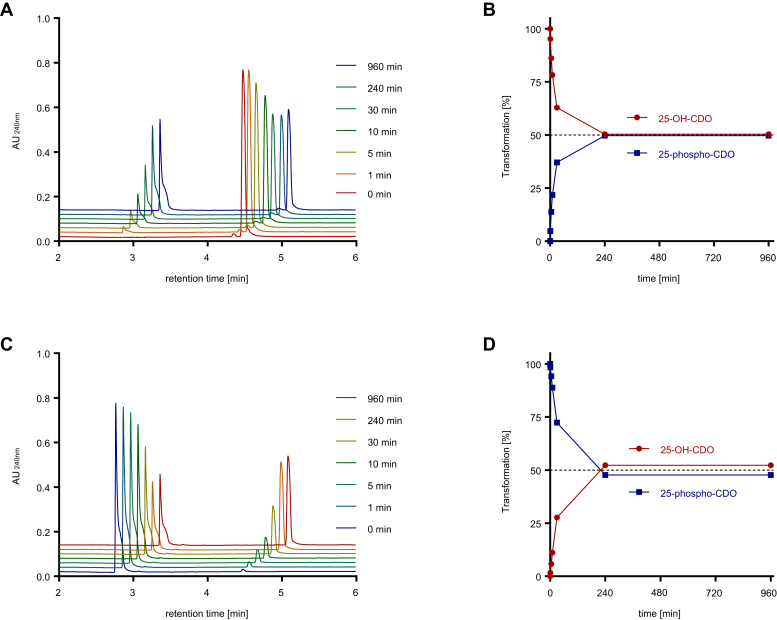
**Conversion of 25-OH-CDO to 25-phospho-CDO with 25-HSK.***A*, UPLC chromatogram of the time-dependent conversion of 1 mM 25-OH-CDO to 25-phospho-CDO by 25-HSK in the presence of 1 mM ATP. *B*, time-dependent conversion of 25-OH-CDO to 25-phospho-CDO. Error bars indicate the standard deviation of three independent replicates. *C*, UPLC chromatogram of the time-dependent conversion of 1 mM 25-phospho-CDO to 25-OH-CDO in the presence of 1 mM ADP. *D*, time-dependent conversion of 25-phospho-CDO to 25-OH-CDO. Error bars indicate the standard deviation of three independent replicates.

Although the ATP concentration was five times higher than that of 25-OH-CDO in the assays, the reaction always levelled off when 70–80% 25-OH-CDO was converted (Fig. S2). To test the possibility whether this observation was due to the establishment of a thermodynamic equilibrium, we followed the reaction at equimolar ATP and 25-OH-CDO concentrations (1 mM each) and observed that the reaction levelled off when 50% of the substrates were converted into the products (Fig. 4, *A* and *B*). A similar result was obtained when the reaction was run in the reverse reaction, thereby following the ADP (1 mM) and 25-phospho-CDO (1 mM) dependent synthesis of ATP and 25-OH-CDO (Fig. 4, *C* and *D*).

**Figure 5:**
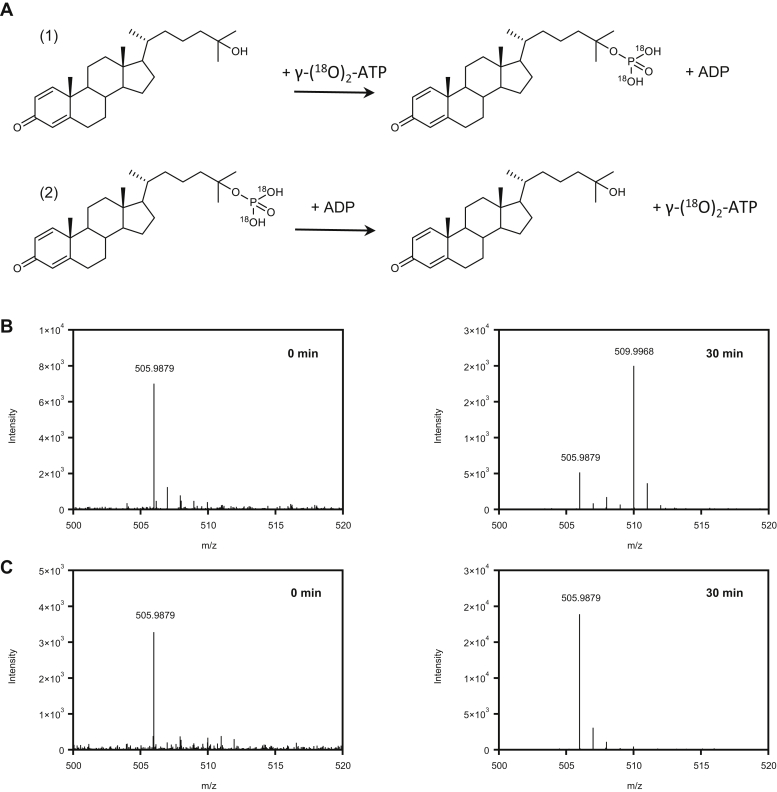
***In vitro* phosphorylation of ADP to ATP using 25-phospho-CDO, or (**^**18**^**O)**_**2**_**-labelled 25-phospho-CDO as phosphoryl donor.***A*, enzymatic production of (^18^O)_2_-labeled 25-phospho-CDO from γ-(^18^O)_2_-ATP in the forward reaction (reaction 1), and the reverse synthesis of γ-(^18^O)_2_-ATP from (^18^O)_2_-labelled 25-phospho-CDO and ADP (reaction 2). *B*, ESI-Q-TOF analysis of the γ-(^18^O)_2_-ATP formed in the presence of (^18^O)_2_-labelled 25-phospho-CDO and ADP after 0 and 30 min incubation. *C*, ESI-Q-TOF analysis of the control experiment with 25-phospho-CDO and ADP.

To verify that the γ-phosphoryl group of the ATP formed in the reverse reaction derives indeed from the 25-phosphoester, we enzymatically synthesized (^18^O)_2_-labeled 25-phospho-CDO using γ-(^18^O)_2_-ATP as phosphoryl donor in the forward reaction, and the (^18^O)_2_-labeled-CDO formed was then reacted with ADP in the reverse reaction (Fig. 5*A*). UPLC electrospray ionization quadrupole-time-of-flight (UPLC-ESI-QTOF) mass spectrometric analysis revealed a mass shift from m/z = 505.9879 (control) to m/z = 509.9968, as expected for γ-(^18^O)_2_-ATP (Fig. 5*B*). In control experiments with nonlabeled 25-phospho-CDO, virtually no γ-(^18^O)_2_-ATP was observed (Fig. 5*C*).

### Kinetic data indicate a ternary complex mechanism and an equilibrium constant near 1

The stoichiometry and kinetics of the 25-HSK catalyzed phosphorylation were determined using the UPLC-based enzymatic assay. In the presence of saturating concentrations of 25-OH-CDO and ATP, a stoichiometry of 1.1 ± 0.1 mol ATP hydrolyzed to ADP per mol 25-OH-CDO formed was determined. In agreement, 25-HSK exhibited only marginal ATPase activity in the absence of 25-OH-CDO (<0.1% of 25-OH-CDO dependent ATPase activity). The kinetic data obtained were evaluated by a fit to Michaelis–Menten curves (Fig. S3), and *K*_*m*_ values of 0.3 ± 0.01 mM for 25-OH-CDO and 0.8 ± 0.05 mM for ATP were calculated for the forward reaction (for a full list of kinetic parameters determined, see Table S3). The reaction was also followed in the reverse direction in the presence of 5 mM ADP and 1 mM 25-phospho-CDO giving *K*_m_ values of 8 ± 0.7 mM for 25-phospho-CDO and 35 ± 3 μM for ADP (Fig. S3). Notably, the high *K*_m_ value for 25-phospho-CDO has to be taken as an apparent value because only 25-phospho-CDO concentrations up to 4 mM (stock concentration: 40 mM in 80% [v/v] 2-propanol/H_2_O) could be tested. At higher concentrations, the added solvent 2-propanol inhibited 25-HSK significantly (at concentrations above 10% [v/v]). As a result, no hyperbolic saturation was reached for the reverse reaction.

The determination of approximately equal equilibrium concentrations of substrate and products (Fig. 4) suggested an equilibrium constant (*K*_*eq*_) of 25-HSK catalyzed reaction near 1. Using the kinetic parameters obtained (Table S3), we determined *K*_*eq*_ according to the Haldane equation (*V*_*f*_, *K*_*mf*_ = forward reaction; *V*_*r*_, *K*_*mr*_ = reverse reaction).

$$K_{eq}=\frac{V_{f}\cdot K_{mr_{25-phospho-CDO}}\cdot {K_{mr}}_{ADP}}{V_{r}\cdot K_{mf_{25-OH-CDO}}\cdot {K_{mf}}_{ATP}}$$

A value of 0.34 was determined, which is in line with the equilibrium concentrations of products and educts determined (Fig. 4*A*).

**Figure 6:**
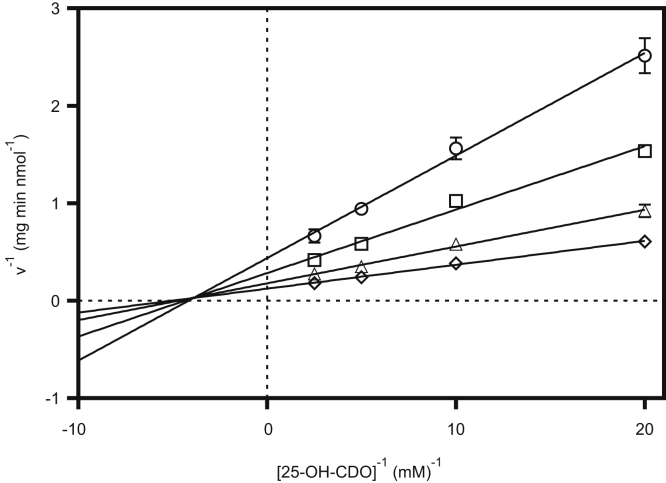
**Double-reciprocal plot of 25-HSK activity *versus* 25-OH-CDO concentration.** The assay was performed by a coupled photometric assay in the presence of 50 (◯), 100 (□), 200 (△), or 400 (◇) μM ATP. Reactions were started with 25-OH-CDO. The error bars represent the standard deviation of three independent experiments.

Reactions catalyzed by kinases usually follow a sequential or a ping-pong mechanism (22). In the former, phosphoryl transfer occurs within ternary complexes between the enzyme and its substrates. Both scenarios can be distinguished for 25-HSK by analyzing rates at varying ATP and 25-OH-CDO concentrations. Double-reciprocal plots of 25-HSK activity *versus* 25-OH-CDO concentrations at four different ATP concentrations yielded clearly intersecting lines, which is consistent with a ternary-complex mechanism (Fig. 6). This result is consistent with the inability to observe a phosphorylated 25-HSK intermediate on SDS-gels that had been preincubated with γ-^32^P-ATP (not shown).

## Discussion

In this work, we identified a highly active kinase that catalyzes the ATP-dependent conversion of 25-OH- to 25-phospho-steroids. This unprecedented phosphorylation of a hydroxyl functionality at a steroid side chain represents a crucial step in the oxygen-independent degradation of zoo- and phytosterols. The identification and isolation of 25-HSK leave little doubt that ATP-dependent dehydration of tertiary 25-OH-steroids to the corresponding Δ24-alkenes is a two-step process involving 25-HSK and a so far unknown C–O lyase that catalyzes the subsequent elimination of phosphate from 25-phospho-CDO. This system differs from the only other known ATP-dependent dehydration system involved in the dehydration of hydrated forms of NAD(P)H (23). The latter reaction plays a role in nicotinamide nucleotide repair and is catalyzed by a single enzyme. Results from kinetic analyses in this work clearly indicate a sequential phosphoryl transfer mechanism of 25-HSK *via* a ternary complex composed of the enzyme, 25-OH-steroid, and MgATP. Such a mechanism has been reported for the majority of kinases (22).

**Figure 7:**
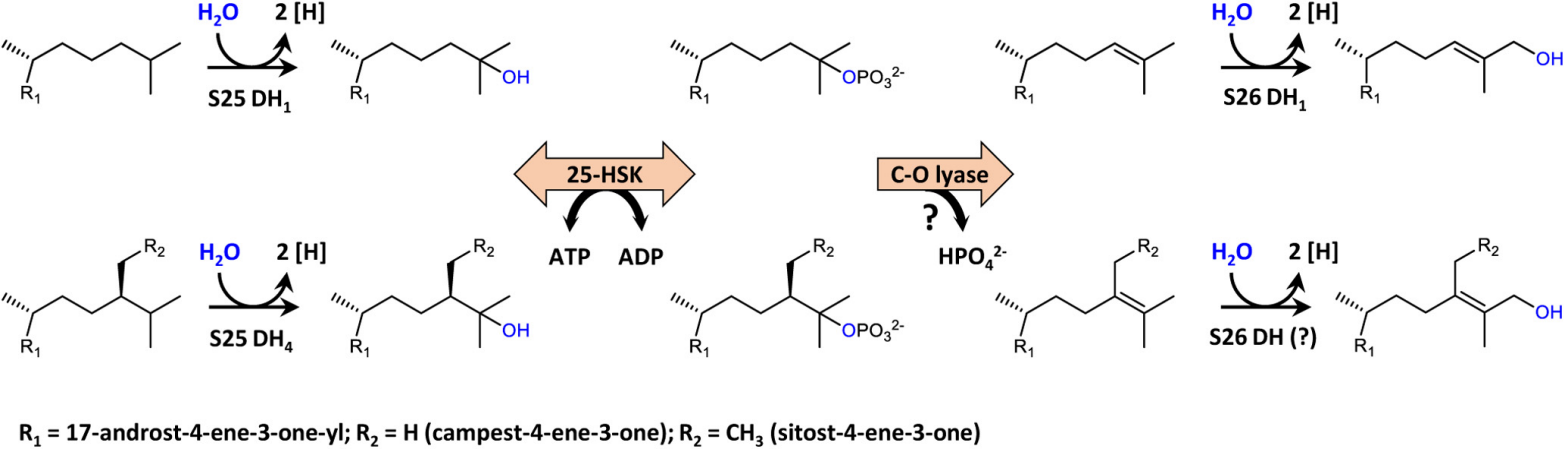
**Initial steps of cholesterol (*****upper panel*****) and sitosterol (*****lower panel*****) side chain degradation in*****S. denitrificans*****.** While specific Mo-dependent hydroxylases exist for the degradation of zoo- and phytosterols (exemplarily shown for cholesterol and sitosterol), only a single promiscuous 25-HSK and, presumably, a so far unknown C–O lyase are involved. For sitosterol, candidate genes for a S26 DH have been proposed (15) but have not been studied, yet.

The anaerobic degradation of zoo- and phytosterols in the model organism *S. denitrificans* involves specific Mo-dependent hydroxylases acting either on tertiary C25 or at allylic C26 (13, 17) (Fig. 7). In contrast, there is only a single gene encoding 25-HSK in the genome of *S. denitrificans*. This finding explains the promiscuity of 25-HSK as evidenced by its ability to phosphorylate 25-OH-steroids with side chain modifications (25-OH-CDO or 25-OH-sitost-1,4-diene-3-one) or with modifications in the sterane skeleton (25-OH-CEO or 25-OH-VitD_3_). Most likely, a promiscuous 25-HSK/25-phosphosteroid lyase tandem accomplishes the ATP-dependent dehydration of various 25-OH-steroids (Fig. 7). The gene encoding the proposed 25-phospho-steroid lyase is unknown, and there is no candidate gene in vicinity of the gene encoding 25-HSK. The phosphorylation of primary 26-OH-steroids is a further promiscuity of 25-HSK, which appears to be rather problematic for the cell because the corresponding 26-phospho-steroids formed are no intermediates during steroid side chain degradation. However, the observed ATP-consuming 26-OH-steroid phosphorylation will be largely prevented *in vivo* by the immediate oxidation of 26-OH-steroid intermediates to the corresponding aldehydes by the 26-OH-steroid forming S26 DHs (15). In contrast to the observed broad substrate spectrum with regard to modification in the isoprenoid side chain or ring A of the sterane skeleton, low-molecular tertiary alcohols such as *tert*-butanol were merely converted (<0.1% of the rate). This finding indicates that the sterane skeleton is essential for specific binding of the tertiary alcohol. Putative gene products with high similarities to 25-HSK from *S. denitrificans* were identified in the denitrifying, estradiol-degrading strain *Denitratisoma* sp. DHT3, though its ability to use side-chain-containing steroids was not reported, yet (Fig. 2) (24). This finding motivated the search for genes encoding putative S25 DHs and S26 DHs, and we indeed identified the corresponding candidate genes in the genome (Table S2). Thus, we propose that *Denitratisoma* sp. DHT3 can degrade side-chain-containing steroids, thereby using the same pathway for steroid side chain degradation as established for *S. denitrificans*. In other denitrifying bacteria that are restricted to the degradation of steroids without side chains such as estradiol (*Denitratisoma oestradiolicus*) (25, 26) or testosterone (*S. denitrificans*) (27, 28), no candidate genes for 25-HSK are present in the genome.

The 25-phosphoester intermediate of anaerobic steroid side chain degradation shows an extremely high phosphoryl group transfer potential, which is in the range of the β,γ-phosphoanhydride of ATP as evidenced by: (i) the establishment of an equilibrium at almost equal substrate/product concentrations, (ii) the establishment of this equilibrium in the forward and reverse reaction, (iii) a *K*_eq_ value of 0.34 as calculated by the Haldane equation using the kinetic parameters determined in this work, and (iv) the formation of γ-(^18^O)_2_-ATP from ADP and the (^18^O)_2_-C25-phosphoester. The standard free Gibbs energy of ATP hydrolysis is around 32 kJ mol^−1^ (29). Our data indicate that 25-phospho-CDO hydrolysis will be similar if not even slightly more negative, around 20 kJ mol^−1^ more negative than standard sugar-phosphate esters (–13 kJ mol^−1^) (29). However, although 25-phosphosteroids can be regarded as an energy-rich cellular molecule with the potential for ATP synthesis, such a reaction will unlikely occur in the cell. During cholesterol degradation, 25-phospho-steroids accumulate only to a very low steady-state concentrations as the subsequent phosphate elimination to the corresponding alkene is considered essentially irreversible (15). The rational for the extremely high phosphoryl group transfer potential of the 25-phosphoesters is likely the highly hydrophobic environment of the double negatively charged phosphoryl-group (at pH 7) that is surrounded by three alkyl functionalities whereas in ATP it is fully hydrated. Other examples of an extremely energy-rich phosphoester in biology are phosphenolpyruvate and poly-phopshorylated inositols. In the former, dephosphorylation allows the unfavorable enol to tautomerize to the much more stable ketone, which renders pyruvate kinase essentially irreversible in the direction of ATP synthesis (30). In poly-phosphorylated inositols, the high electrostatic repulsion increases the Gibbs free energy of hydrolysis in comparison to sugar-monophosphates. Indeed, reversibility of inositol phosphate (hydroxyl) kinases has been experimentally shown (31), albeit with *K*_eq_ values of 14 being clearly higher than that of 25-HSK (32).

## Experimental procedures

### Chemicals and bacterial strains

The chemicals used in this work were of analytic grade. *S. denitrificans* Chol-1S (DSMZ 13999) was obtained from the Deutsche Sammlung für Mikroorganismen und Zellkulturen (DSMZ). *E. coli* BL21 (DE3) and *E. coli* 5α were obtained from New England Biolabs (NEB).

### Synthesis of sitost-4-en-3-one and 26-OH-CEO

(25R)-26-OH-cholesterol was chemically synthesized (33) and kindly provided by Prof N. Breit. Sitost-4-en-3-one or 26-OH-cholest-4-en-3-one was enzymatically synthesized from commercially available β-sitosterol or (25R)-26-hydroxycholesterol using 50 mM MOPS/KOH buffer (pH 7.0), 9% (w/v) 2-hydroxypropyl-β-cyclodextrin (HPCD), 2 mM NAD^+^, 2 mM MgCl_2_, 1 mM β-sitosterol, cholesterol oxidase and catalase as described before (15). The enzymatic synthesis was performed for 16 h. The product was extracted with two volumes ethyl acetate, evaporated (240 mbar, 40 °C), and purified *via* a preparative HPLC column (XSelect, CSH prep C18 5 μM OBD 19 × 250 mm) using a water/2-propanol gradient from 15% to 100% 2-propanol (5 ml min^−1^). The product was collected, lyophilized, and dissolved in 2-propanol to a final volume of 50 mM.

### Synthesis of 25-OH-steroids

25-OH-cholest-1,4-diene-3-one (25-OH-CDO) was enzymatically synthesized from commercially available cholest-4-en-3-one (CEO) using the AcmB gene product and S25 DH_1_ from *S. denitrificans* after heterologous production in *E. coli* Bl21 (DE3) and *Thauera aromatica* K172 as described before (15). For synthesis of 25-OH-sitost-1,4-diene-3-one, S25 DH_1_ was replaced by S25 DH_4_, and for synthesis of the 4-en-3-one moiety of steroids, AcmB was omitted. The enzymatic reaction mixture contained 2 mM substrate, 50 mM MOPS/KOH buffer (pH 7.5), 9% (w/v) HPCD, 5 mM K_3_[Fe(CN)_6_], 2% (v/v) volume crude extracts from *E. coli* Bl21 producing AcmB, and/or 3% (v/v) volume crude extracts from *T. aromatica* K172 producing S25 DH_1_ or S25 DH_4_, respectively. The enzymatic synthesis was performed in 100 ml at 30 °C for at least 16 h until the substrate was completely transformed to the 25-OH-product. Extraction and purification were performed as described above.

### Synthesis of 25-phospho-CDO

25-phospho-CDO was enzymatically synthesized from 25-OH-CDO using 25-hydroxy-steroid kinase (25-HSK) overproduced in *E. coli* BL21 (DE3). The enzymatic reaction mixture contained 1 mM 25-OH-CDO, 50 mM Tris/HCl buffer (pH 7.8), 9% (w/v) HPCD, 2 mM ATP, 10 mM MgCl_2_, and 25 μg ml^−1^ 25-HSK. The synthesis was performed in 100 ml scale at 30 °C for 30 min. The products were extracted and separated as described above. The lyophilized product was dissolved in 80% (v/v) 2-propanol/water to a final volume of 40 mM.

### Culture conditions and preparation of cell extracts

*S. denitrificans* was cultivated under denitrifying conditions in a phosphate-buffered medium (3 g L^−1^ NaH_2_PO_4_ × 2 H_2_O, 4 g L^−1^ K_2_HPO_4_, 0.54 g L^−1^ NH_4_Cl at pH 6.9) at 30 °C with 2 mM cholesterol as carbon source in a 200-L-fermenter. Cells were harvested in the late exponential phase (8000*g*, 20 min, and 4 °C) and frozen in liquid nitrogen. Frozen cells were lysed by a French pressure cell at 137 MPa in the presence of two volumes of lysis buffer containing 50 mM MOPS/KOH (pH 7.0) and 0.1 mg DNase I.

### Enzyme assays

The ATP-dependent phosphorylation of 25-OH-CDO to 25-phospho-CDO was measured at 30 °C (750 rpm) in the presence of 50 mM Tris/HCL buffer (pH 7.8), 1 mM 25-OH-CDO, 5 mM ATP, 10 mM MgCl_2_, 9% (w/v) HPCD, and 5–50 μg ml^−1^ 25-HSK unless otherwise stated. Enzyme assays (50 μl) were stopped by adding four volumes 2-propanol. For detection of steroid intermediates *via* UPLC, the mixture was centrifuged twice at 8000*g* (4 °C). The supernatant was loaded on an Acquity H-class UPLC system (Waters). An Acquity UPLC CSH C18 column (1.7 μM, 21 × 100 mm) was used for separation with a 5–100% acetonitrile gradient (40 °C) in aqueous NH_4_OAc (10 mM) at a flow rate of 0.35 ml min^−1^.

The ADP-dependent reverse reaction was followed at 30 °C (750 rpm) in the presence of 50 mM Tris/HCl (pH 7.8), 1 mM 25-phospho-CDO, 5 mM ADP, 10 mM MgCl_2_, 9% (w/v) HCPD, and 5–50 μg ml^−1^ 25-HSK unless otherwise stated. The enzyme assays were prepared for separation *via* UPLC as described above. The kinetic parameters were determined *via* UPLC-based enzymatic assays by varying one substrate concentration at a fixed saturating concentration of the other. For 25-OH-CDO, 5 mM ATP and for 25-phospho-CDO, 5 mM ADP was added to determine kinetic parameters. For ATP or ADP, 1 mM 25-OH-CDO or 1 mM 25-phospho-CDO was added, respectively.

For determination of nucleotides (ATP/ADP), enzymatic assays (20 μl) were stopped with 5 μl 0.25 M HCl and centrifuged (15 min, 8000*g*, 4 °C). Then, 10 μl of the supernatant was transferred to 190 μl 10 mM tri-methyl-ammonium acetate (TEAA) buffer pH 7.0. For detection, samples were applied to an Acquity H-class UPLC system (Waters) using an Acquity UPLC HSS T3 (1.8 μM, 2.1 × 100 mm) column. Nucleotides were separated with a gradient of 0–10% acetonitrile in 10 mM TEAA buffer at a flow rate of 0.4 ml min^−1^.

For the coupled photometric assays, the following setup was used: 50 mM Tris/HCl (pH 7.8), 9% (w/v) HPCD, 5 mM ATP, 10 mM MgCl_2_, 0.25 mM NADH, 1 mM phosphoenolpyruvate, 10 U myokinase, 10 U pyruvate dehydrogenase, 10 U lactate dehydrogenase, 0.5 mM of substrate, and 5–1000 μg ml^−1^ 25-HSK. The enzyme activity was followed by the coupled ATP hydrolysis to the reduction of NADH to NAD^+^ at 365 nm (ε = 3.4 × 10^−3^ mol^−1^ cm^−1^) at 25 °C.

### Enrichment of 25-HSK from cholesterol grown *S. denitrificans*

After ultracentrifugation (150,000*g*, 1.5 h, and 4 °C) of crude extracts from *S. denitrificans*, the soluble protein fraction was applied to a DEAE-Sepharose column (60 ml, GE Healthcare) at 5 ml min^−1^ with buffer A_1_ (20 mM Tris/HCl [pH 7.8]). The active protein fraction eluted by increasing the amount of buffer A_2_ (20 mM Tris/HCl [pH 7.8] and 500 mM KCl) from 30% (150 mM KCl) to 40% (200 mM KCl). Active fractions were concentrated using a 10-kDa cutoff membrane, desalted with PD-10 columns (Sephadex G-25 M), and diluted in ten volumes buffer B (20 mM Tris/HCL [pH 7.8] and 1 M (NH_4_)_2_SO_4_). The concentrated fraction was applied to an Octyl-Sepharose column (15 ml, GE Healthcare) at 3 ml min^−1^. Active fractions were obtained by increasing the amount of buffer A_1_ from 90% (corresponding to 100 mM [NH_4_]_2_SO_4_) to 100% (corresponding to 0 mM [NH_4_]_2_SO_4_). Active fractions were concentrated, diluted in ten volumes buffer C (20 mM HEPES/KOH, pH 7.6, and 150 mM KCl), and applied to a Superdex 200 Increase 10/300 GL column at a flow rate of 0.5 ml min^−1^.

### Enrichment of recombinant 25-HSK_Strep_ from *E. coli* BL21

After ultracentrifugation (150,000*g*, 1 h, 4 °C) of crude extracts from *E. coli* BL21, the soluble protein fraction was applied to a Strep-Tactin affinity column (20 ml, GE Healthcare) at 2 ml min^−1^. The column was equilibrated with buffer D_1_ (50 mM HEPES/KOH (pH 7.6), 150 mM KCl). The active protein fraction eluted with buffer D_2_ (50 mM HEPES/KOH, pH 7.6, 150 mM KCl, and 5 mM desthiobiotin). The enriched protein was concentrated (10-kDa cutoff membrane), desalted (PD-10 column), and stored at 4 °C (>50 mg mL^−1^) or frozen in liquid nitrogen for long-term storage.

### Protein identification by mass spectrometry of peptides

Proteins were identified by excising the bands of interest from a SDS-PAGE gel. The bands were washed 3× with 80 μl 10 mM NH_4_HCO_3_ (10 min) and 80 μl 50% EtOH in 5 mM NH_4_HCO_3_ (10 min). The gel bands were incubated with 80 μl 10 mM DTT in 10 mM NH_4_HCO_3_ (30 min, 56 °C), the supernatant was removed, and 80 μl 50 mM iodoacetamide in 10 mM NH_4_HCO_3_ was added (30 min, 25 °C). The supernatant was removed and the bands were washed 3× with 10 mM NH_4_HCO_3_ (10 min) and 100% EtOH, and then dried with a Speedvac Vacuum Concentrator (60 min, 40 °C). The dried gel bands were incubated with 20 μl trypsin at 37 °C (0.6 μg). The proteins were released from the gel bands by adding 75 μl 0.05% TFA in 50% acetonitrile. The samples were sonicated for 10 min and afterward dried *via* a Speedvac Vaccum Concentration (120 min, 45 °C). The dried products were dissolved in 20 μl 0.1% TFA and applied to an Acquity UPLC I-class system (Waters) using an Acquity UPLC peptide CSH C18 column (1.7 μm, 2.1 mm × 150 mm). For separation, a gradient of 40% acetonitrile + aqueous 0.1% formic acid at a flowrate of 40 μl min^−1^ was used. Proteins were identified using a Synapt G2-Si high-resolution ESI-Q-TOF mass spectrometry system (Waters) operating in positive mode at 3 kV capillary voltage, 350 °C desolvation temperature, and a desolvation gas flow of 800 L h^−1^. The resulting spectra were analyzed with ProteinLynx Global Server 3.0.3 (Waters) by matching with the UniProt database of *S. denitrificans* (3020 protein-coding sequences). The following parameters were used for protein identification *via* ProteinLynx Global Server: Mass tolerance for precursor ions = 5 ppm, mass tolerance for fragment ions = 13 ppm, minimal fragments per peptide = 3, minimal peptides per protein = 1, minimal fragments per protein = 7, false discovery rate = 4%.

### LC-ESI-MS analysis of steroid compounds

Metabolites were analyzed by an Acquity I-class UPLC system (Waters) using an Acquity UPLC CSH C18 (1.7 μm, 2.1 × 100 mm) column coupled to a Synapt G2-Si HRMS ESI-Q-TOF device. For separation, an aqueous 10 mM ammonium acetate/acetonitrile gradient was used as described above (UPLC). Samples were measured in positive mode with a capillary voltage of 2 kV, 100 °C source temperature, 450 °C desolvation temperature, 1000 L min^−1^ N_2_ desolvation gas flow, and 30 L min^−1^ N_2_ cone gas flow. The evaluation of the data was performed using the MassLynx 4.1 (Waters) software. Metabolites were identified by their retention times and *m/z* values.

### Heterologous gene expression and production of 25-HSK_Strep_ in *E. coli* BL21

The gene encoding 25-HSK (SDENChol_21286) was amplified with a C-terminal Strep-tag by PCR from *S. denitrificans* genomic DNA as the template using the following primers: 21286_for (GTCATGGCTAGCATGTCTGATACACCGGATCTG) and 21286_rev (CTAGTCAAGCTTTTACTTTTCGAACTGCGGGTGGCTCCATCCTCCGAAATCCCAGGGATCTTCGC). The resulting 1.2 kb DNA fragment was NheI/HindIII double digested, cloned into pASK-IBA15plus, and transformed into *E. coli* BL21 according to protocols from New England Biolabs (NEB). Induction of 25-HSK was carried out in 2× YT medium (18 g L^−1^ tryptone, 10 g L^−1^ yeast extracts, and 5 g L^−1^ NaCl) supplemented with 100 μg ml^−1^ ampicillin. When optical density at 600 nm reached values above 0.5, 20 μg ml^−1^ anhydrotetracycline (AHT) was added. Induction was carried out at 20 °C while shaking at 180 rpm. Cells were harvested in the late exponential phase (>16 h induction) by centrifugation (8000*g*, 20 min, and 4 °C), and frozen in liquid nitrogen.

### Synthesis γ-(^18^O)_2_-ATP

γ-(^18^O)_2_-ATP was synthesized as described previously (34) (for a scheme of synthesis and structures of chemicals used, see Fig. S4). In brief, a P-amidite **3** was generated by hydrolysis of benzylbromide **1** with ^18^O labeled water (isotopic purity >98%). Benzylic alcohol **2** was reacted with 0.5 equivalents of di*iso*propylphosphoramidous dichloride giving P-amidite **3**. The P-amidite **3** was used for the introduction of two terminal ^18^O labels by reaction with ADP (tetrabutylammonium salt in DMF as solvent), followed by oxidation of the P(III)-P(V) mixed anhydride with *m*CPBA. The crude material was precipitated from solution by addition of diethylether and the benzyl protecting groups were removed by hydrogenation over Pd/C at elevated pressure (5 bar). The crude material was purified by chromatography over DEAE Sepharose (elution with 30–100 mM aq. NH_4_HCO_3_). Excess buffer was removed by repeated freeze drying giving pure ^18^O labeled ATP as ammonium salt. The ammonium salt was passed through a Dowex-H^+^ column and aq. NaOH solution (2.0 eq.) was added. After lyophilization, the desired heavy ATP was obtained as a colorless solid. Mass spectrometry revealed high isotopic enrichment in the product (>97%).

## Data availability

All data are available upon request. The protein mass spectrometry data have been deposited to the ProteomeXchange Consortium *via* the PRIDE (https://doi.org/10.1093/nar/gky1106) partner repository with the dataset identifier PXD027031 and 10.6019/PXD027031.

## Supporting information

This article contains supporting information (34).

## Conflict of interest

The authors declare that they have no conflicts of interest with the contents of this article.
