# Supplementary material for: A fully reversible 25-hydroxy steroid kinase involved in oxygen-independent cholesterol side-chain oxidation
Source: J Biol Chem. 2021 Aug 21;297(4):101105. doi: 10.1016/j.jbc.2021.101105 (PMC8449060; doi:10.1016/j.jbc.2021.101105)
Supplement: Table S1–S3 and Figures S1–S4 [file mmc1.docx]

**A fully reversible 25-hydroxy steroid kinase involved in oxygen-independent cholesterol side-chain hydroxylation**

Christian Jacoby^1^, Malina Goerke^1^, Dominik Bezold^2^, Henning Jessen^2^, Matthias Boll^1^*

From the ^1^Faculty of Biology, Albert-Ludwigs-Universität Freiburg, Freiburg, Germany,

^2^Institute of Organic Chemistry, Albert-Ludwigs-Universität Freiburg

­­­­­­­­­­­­­­­­___________________________________________________________________________

**Supplementary Information**

Supplementary Tables S1-S3

Supplementary Figures S1-S4

**Table S1. ESI-Q-TOF analyses of tryptic digests from protein bands obtained during enrichment of 25-HSK from cholesterol grown *S. denitrificans*.** Sequence coverages with a Score ≥ 1000 matching with the UniProt database of *S. denitrificans* are shown. In bold, the gene encoding for a phosphotransferase-like protein is shown, that was predicted to code for 25-HSK.

| Mass of excised SDS protein band | Gene product  identified | Accesion # Uniprot | Score | Matched peptides | Sequence coverage | Mass deduced from amino acid sequence |
| --- | --- | --- | --- | --- | --- | --- |
| 60 | SDENChol_11189 | A0A7Z7HR48 | 2351 | 27 | 44.4% | 59 |
| 50 | SDENChol_10468 | A0A7Z7HP62 | 4824 | 24 | 57.4% | 53 |
| 40 | **SDENChol_21286**  SDENChol_20263 SDENChol_10881  SDENChol_10869  SDENChol_10175 | **A0A7Z7HU23**  A0A7Z7HRV5  A0A7Z7MUQ9  A0A7Z7HPU6  A0A656Z6Q0 | **7371**  2427  2407  2406  2317 | **41**  14  16  16  11 | **66.5%**  61.3%  54.3%  54%  37.3% | **43**  39  43  44  40 |
| 30 | SDENChol_10757 | A0A656Z7J7 | 3295 | 12 | 49.1% | 33 |

**Table S2. Amino acid sequence identities of 25-HSK, S25 DH_1_ and S26 DH_1_ from *S. denitrificans* with predicted gene products of *Denitratisoma* species.** Protein blast analysis were performed via the NCBI database (refseq _protein). Genes with sequence identities > 50% are shown.

|  | **species** | | |
| --- | --- | --- | --- |
| **Protein function** | ***S. denitrificans***  **[SDENChol_XXXXX]** | ***Denitratisoma sp.* DHT** | ***Denitratisoma oestradiolicum*** |
| Catalytic α-subunit S25DH_1_ | 20804  (WP_154717222.1) | 73.41%  (WP_145842274.1) | 73.72%  (WP_145771531.1) |
| 25-HSK | 21286 (WP_154717265.1) | 55.26% (WP_186453974.1)  50.68% (WP_145841310.1) | - |
| Catalytic α-subunit S26DH_1_ | 10534  (WP_154715926.1) | 77.65%  (WP_145840597.1)  68.34% (WP_145840458.1) | 76.07%  (WP_145769701.1)  66.84% (WP_145771097.1) |

**Table S3. Kinetic parameters of 25-HSK from *S. denitrificans*.** The forward and reverse maximal specific activities were determined with enriched 25-HSK in three independent experiments. The equilibrium constant (*K_eq_*) was calculated using equation (Eqn 1). *K*_mf_,*V*_f_ = *K*_m_, *V*_max_ forward reaction, *K*_mr_*,V*_r_ = *K*_m_, *V*_max_ reverse reaction.

| **Parameter** | **Fitted value** |
| --- | --- |
| $V_{f} (s^{-1}$) | 18 ± 0.2 |
| $V_{r} (s^{-1})$ | 60 ± 4 |
| ${K_{mf}}_{25-OH-CDO}(mM)$ | 0.3 ± 0.01 |
| ${K_{mr}}_{25-phospho-CDO}(mM)$ | 8 ± 0.7 |
| ${K_{mf}}_{ATP}(mM)$ | 0.8 ± 0.05 |
| ${K_{mr}}_{ADP} (mM)$ | 0.035 ± 0.003 |
| $K_{eq} (mM)$ | 0.34 |

**Figure S1. Transformation of 25-OH-CDO in crude S. denitrificans extracts and in active pools obtained after DEAE-Sepharose-, and Octyl-Sepharose chromatography.** The transformation of **1** 25-OH-CDO to **4** 26-OH-DDO via **2** 25-phospho-CDO, and **3** DDO is shown. In the pools after DEAE- and Octyl-Sepharose chromatography, only formation of **2** was observed.

**Figure S2. Time-dependent conversion of 25-OH-CDO to 25-phospho-CDO using recombinant 25-HSK.** The conversion of 1 mM 25-OH-CDO and 5 mM ATP was followed with 10 µg mL^–1^ 25-HSK via UPLC based enzymatic assays. The mean values ± standard deviation of three independent replicates are shown.

**Figure S3. Kinetic characterization of recombinant 25-HSK.** Saturation of enzyme activity is shown for 25-OH-CDO (A, in the presence of 5 mM ATP), for ATP (B, in the presence of 1 mM 25-OH-CDO) in the forward direction; for 25-phospho-CDO (C, in the presence of 5 mM ADP) and for ADP (D, in the presence of 1 mM 25-phospho-CDO) in the reverse direction. The error bars represent the standard deviation of three independent experiments.


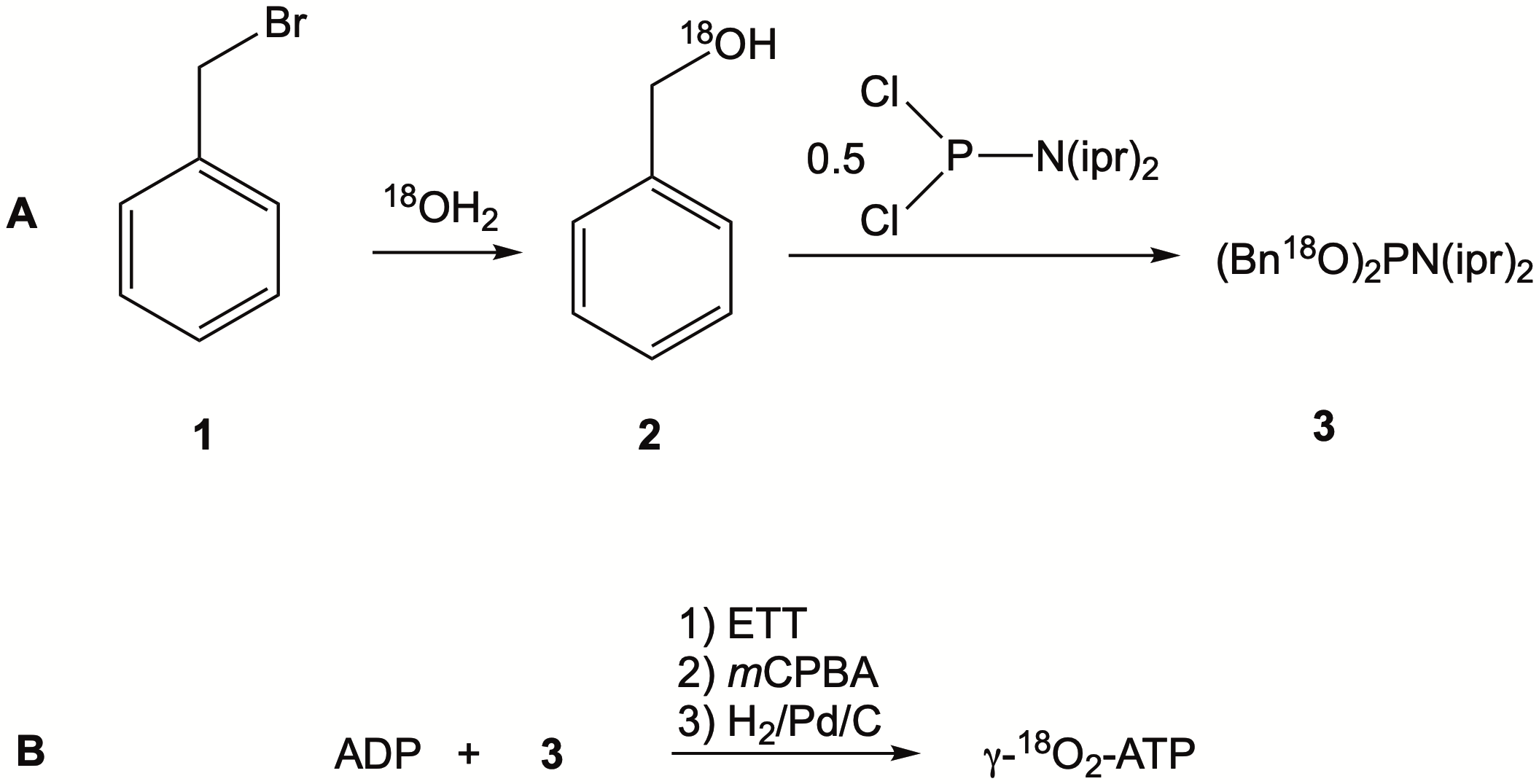


**Figure S4.** *A*, Synthesis of P-amidite **3** for the introduction of ^18^O labels into phosphoric anhydrides. *B*, Application in the synthesis for stable isotope labeled ATP. Abbreviations: ETT ethylthiotetrazole, *m*CPBA *meta-*chloroperbenzoic acid. Synthesis was according to the procedure described by Hofer et al. 2015 (34).
